# Micro-Ultrasonic Viscosity Model Based on Ultrasonic-Assisted Vibration Micro-Injection for High-Flow Length Ratio Parts

**DOI:** 10.3390/polym12030522

**Published:** 2020-03-01

**Authors:** Yan Lou, Jianjun Xiong

**Affiliations:** Guangdong Provincial Key Laboratory of Micro/Nano Optomechatronics Engineering, College of Mechatronics and Control Engineering, Shenzhen University, Shenzhen 518060, China; Xjianjun01@163.com

**Keywords:** micro-injection, MU viscosity model, ultrasonic energy, characteristic microdimension, accuracy

## Abstract

A micro-ultrasonic (MU) viscosity model based on ultrasonic-assisted vibration micro-injection for high- flow length ratio polymer parts was established. This model considered the effects of ultrasonic energy and the characteristic microdimension. Ultrasonic energy parameters (such as the ultrasonic amplitude, frequency, and ultrasound velocity), the characteristic microdimension, and the molecular chain length (MCL) were introduced into the MU viscosity model. An ultrasonic micro-injection experimental platform was built on an injection molding machine. Polypropylene (PP) filling experiments were carried out using microgrooves with different flow length ratios (depth-to-width ratios of 3:1, 5:1, and 10:1). The validity and accuracy of the MU viscosity model were examined through a filling experiment with polypropylene (PP) microgroove injection molding and by a flow pressure difference experiment with polystyrene (PS). The results showed that the MU viscosity model was in better agreement with the experimental results compared to other models. The maximum error of the MU model was 4.9%. Ultrasound-assisted vibration had great effects on the filling capacity for microgrooves with high flow length ratios (depth-to-width ratios greater than 5:1). The filling capacity increased as the ultrasonic amplitude increased.

## 1. Introduction

With the development of micro-electro-mechanical systems (MEMS), the development of micro-structures is becoming more and more demanding, and the application value of the high-flow length ratio parts market is gradually reflected. At present, the processing methods of high-flow length ratio microstructure parts include LIGA [1], UV-LIGA [2], deep reflection ion etching [3], and micro-injection molding [4]. Because of its low cost and high efficiency, micro-injection molding can be continuously used in the processing and molding of high flow length ratio parts.

However, as the flow length ratio increases, the heat dissipation rate of the melt accelerates, resulting in faster melt temperature drop, increased viscosity, reduced polymer flow ability, and shrinkage warpage, welding defects and so on. In order to solve the defects of high flow length ratio injection parts, there are two main research directions at present, one is to optimize the micro-injection process parameters, such as mold temperature, polymer melt temperature, injection pressure, injection speed, etc. [5,6,7]; the other is to change the external conditions to increase the molding quality, such as gas-assisted in the mold system, vacuum or external ultrasonic energy field for micro injection [8].

Ultrasonic processing technology, as a novel molding processing technology, has been widely used in recent years for the injection molding, extrusion molding, and compression molding of polymers [9,10]. Despite different ways of applying ultrasonic energy, studies have found that ultrasounds can affect the microstructure filling performance [11], change the material properties [12], affect the extrusion swelling [13], improve the weld line strength of the plastic parts [14], and reduce the interface friction during ejection [15]. However, current studies are mainly based on experiments. There is little research on the influence of ultrasonic energy on the rheological properties of polymers. Isayev [16] analyzed the relationship between the shear rate and viscosity for low-density polyethylene (LDPE), polypropylene (PP), and polystyrene (PS) under different low-frequency oscillations. Gao [17] analyzed the mechanism of longitudinal ultrasonic-assisted microinjection molding (LUμIM). The longitudinal ultrasonic vibration energy was divided into transmission energy and conversion energy. A viscosity model under ultrasonic energy was established based on the Power-Law viscosity model and considering the interaction between the two types of energies. However, the validity and accuracy of the model were not examined. Chen [13] designed an ultrasonic vibration extruder based on the principle of viscosity measurements by a capillary rheometer. The polymer viscosity under ultrasonic energy was measured. The data was fitted to obtain the viscosity expression under ultrasonic energy: $\ln\eta =\frac{E_{a}}{kE_{U}}+\ln A$, where η is the viscosity, $E_{a}$ is the viscous flow activation energy, *k* is a coefficient, *E_U_* is the ultrasonic energy, and *A* is a parameter. However, the model does not consider the influence of the structural characteristic dimension on the viscosity during microflow.

Based on the macroscopic Power-Law viscosity model, this paper established a MU viscosity model that can reflect the flow characteristics under ultrasonic energy. The effects of ultrasonic vibration energy, the molecular chain length (MCL), and the characteristic dimension of microparts on viscosity were considered. The validity and accuracy of the model were examined through a filling experiment with polypropylene (PP) microgroove injection molding and by a flow pressure difference experiment with polystyrene (PS). This model can better predict the flow characteristics of micro injection molding polymers under ultrasonic vibration conditions.

## 2. Establishment of MU Viscosity Model

Based on the power-law viscosity model (Equation (1)),

(1) $$\eta =K{\dot{\gamma }}^{n-1}$$

where η is the viscosity, MPa.s; *K* is the consistency index, Pa.s; $\dot{\gamma }$ is the shear rate, /s; and *n* is the non-Newtonian index.

The relationships of the consistency index *K* and the non-Newtonian index *n* with the ultrasonic vibration energy *Eu* can be established. The experimental data of ultrasonic-assisted vibration extrusion in reference [13] showed that the higher the ultrasonic energy *Eu*, the smaller the consistency index *K*, and the greater the non-Newtonian index *n*. Therefore, the ultrasonic energy has linear relationships with ln*K* (consistency index) and the non-Newtonian index *n*. Equations (2) and (3) are thus obtained. The model parameter values are obtained by data fitting.

(2) $$lnK=a_{1}+b_{1}\times E_{u}$$

(3) $$n=a_{2}+b_{2}\times \frac{1}{E_{u}}$$

where *a*_1_ and *b*_1_ are consistency index correction parameters, *a*_2_ and *b*_2_ are non-Newtonian index correction parameters, which can be obtained through experiments. *E_u_* is the ultrasonic vibration energy (W). *E_u_* can be obtained as $E_{U}=2\pi^{2}\rho uf^{2}A^{2}S\alpha$, where *f* is the ultrasonic frequency (Hz), *A* is the ultrasonic amplitude (μm), *S* is the ultrasonic active area (m^2^), *u* is the propagation velocity of the ultrasonic waves in the material (m/s), *ρ* is the material density (kg/m^2^), and α is the sound absorption coefficient of the material.

Substituting Equations (2) and (3) into the viscosity model (Equation (1)) to obtain the ultrasonic viscosity model (Equation (4)) gives:

(4) $$\eta =e^{\left(a_{1}+b_{1}\times Eu\right)}\times {\dot{\gamma }}^{\left(a_{2}+b_{2}\times \frac{1}{Eu}-1\right)}$$

When the characteristic dimension of the microchannel is reduced, the movement space of the macromolecular chains is reduced, the macromolecular chains and chain segments move more significantly along the flow direction, and the viscosity of the melt is reduced [18]. Based on the study in reference [19], correction parameters based on the characteristic dimension and the MCL are introduced into Equation (4). The microviscosity model under ultrasonic energy is

(5) $$\eta_{micro}=\left(a_{3}\times \frac{l_{e}}{d}+b_{3}\right)\times e^{\left(a_{1}+b_{1}\times E_{u}\right)}\times {\dot{\gamma }}^{\left(a_{2}+\frac{b_{2}}{E_{u}}-1\right)}\;E_{U}=2\pi^{2}\rho uf^{2}A^{2}S\alpha$$

where *a*_3_ and *b*_3_ are the microdimension correction parameters, which can be obtained through experiments. *le* is the MCL, and *d* is the characteristic dimension of the microstructure.

## 3. Experiments and Simulation

### 3.1. Experiments Device and Method

**Figure 1:**
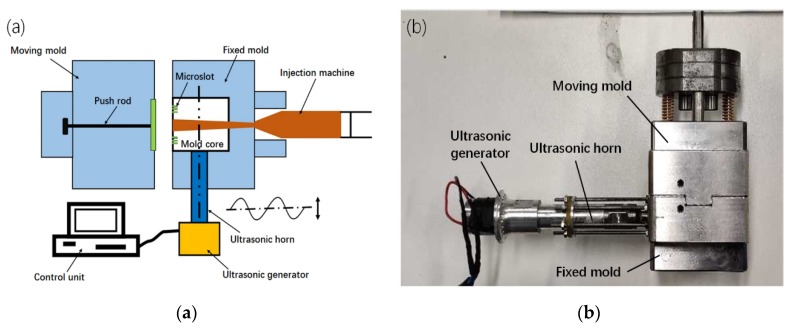
The ultrasonic microinjection molding platform (**a**) schematic diagram (**b**) physical map.

The ultrasonic-assisted vibration micro-injection experiment was performed on a Babyplast microinjection machine (Babyplast-6/10P, Babyplast, Molteno, Italy). The ultrasonic equipment consisted of an ultrasonic transmitter and an ultrasonic horn. The vibration frequency of the ultrasonic generator (Ivyson, Shenzhen, China) is 38.9 KHz. A schematic diagram of the ultrasonic-assisted vibration microinjection molding platform is shown in Figure 1. Ultrasonic vibration was directly applied to the mold core in the form of transverse waves. The ultrasound amplitude was measured on a CMOS laser displacement measuring device (LK-G5001, KEYENCE, Osaka, Japan). The characteristic dimension of the microstructure was measured with a laser confocal microscope (VK-250, KEYENCE, Osaka, Japan).

Prior to microinjection, the polypropylene (PP, 2401, Yanshan Petrochemical, Beijing, China) was dried in a vacuum drying oven (DZF-6050, Subo Instruments, Shaoxing, China) at 60 °C for 4 h.

#### 3.1.1. Rectangular Microgroove Structure of the Mold

**Figure 2:**
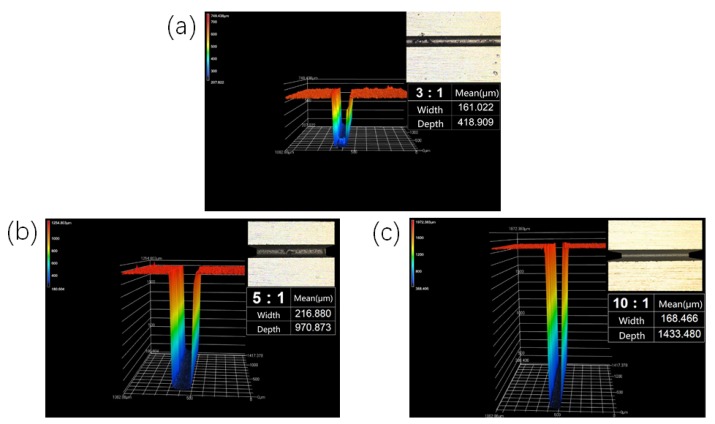
Actual dimensions of the mold microgrooves (**a**) flow length ratio 3:1; (**b**) flow length ratio 5:1; (**c**) flow length ratio 10:1.

The mold was fabricated on a slow-feeding wire-cut machine (AP250LS, Sodick, Kanagawa, Japan). The design dimensions of the microgrooves are shown in Table 1. The actual widths (on top of microgroove) and depths of the microgrooves (averaged after five measurements) were measured with a laser confocal microscope. The results are shown in Table 1 and Figure 2. The actual dimensions of the microgrooves were used for the calculation of the filling capacity in Section 3.2 below.

#### 3.1.2. Ultrasonic Micro-Injection Experiment

The experimental parameters are shown in Table 2. A series of experiments of ultrasonic micro-injection were carried out to investigate the effect of processing parameters on filling capacity of PP. The processing parameters include injection pressure, injection speed, and ultrasound amplitude. Therefore, we conducted three groups of experiments (Group A, B, C) with only one variable to explore the influence of each parameter, and the variables and their values are listed in Table 2. The temperatures of PP melt and mold body were set as 200 °C and 25 °C, respectively. To achieve a steady and accurate filling capacity, experiments with the same parameters were conducted for five times and five results obtained were averaged as the final filling capacity under certain parameters. The height of the rectangle injection molded part was measured with a laser confocal microscope (averaged after five measurements) and then compared with the depth of the microgroove of the mold to obtain the filling capacity. The filling capacity can be obtained as $f=(H_{S}/H$)×100%, where *H_s_* is the average value of the height of the rectangle injection molded part, and  H is the depth of the microgroove of the mold.

### 3.2. Simulation Modeling

To validate the MU viscosity model established in this study, two finite element models of the ultrasonic-assisted vibration micro-injection process were built in commercial analysis software Fluent: (1) simulation of microstructure filling capacity with PP material under ultrasound; (2) pressure difference simulation with PS material under ultrasound.

#### 3.2.1. Simulation of Filling Capacity

**Figure 3:**
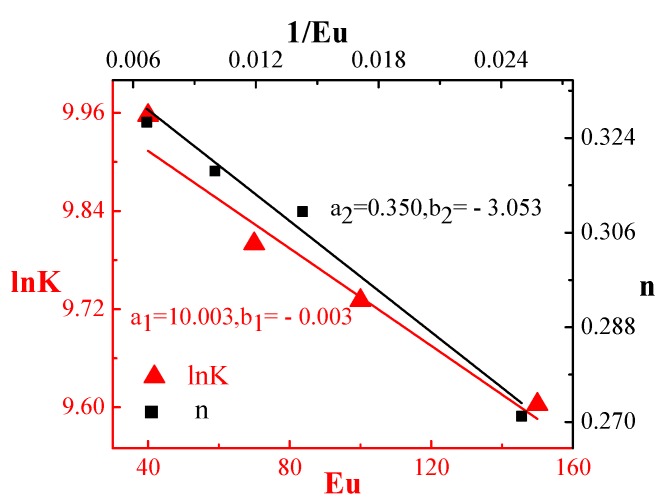
Linear relationships of *lnK* (consistency index) and non-Newtonian index *n* with ultrasonic energy *Eu.*

PP was selected as the research material. Equation (5) was imported into the preprocessing module of simulation software FLUENT as a user-defined function to describe the polymer viscosity. One short edge of the model was set as inlet and the other three edges were set as mold walls. The ultrasonic frequency f = 38.9 KHz, the amplitude A = 19.55 μm. The flow length ratios of the rectangular microgrooves were 3:1, 5:1, and 10:1, as shown in Table 1, and *d* were set as 161.002 μm, 211.839 μm and 171.142 μm, respectively. To compare with the experiments, the dimensions of the rectangular microgroove used in the simulation were consistent with those in the experiments (Figure 2). The injection pressures were set to 6, 7, 8, 9, and 10 MPa, the injection speed was 40 mm/s, and the mold temperature was 25 °C. When the temperature of melted PP is 200 °C, the linear relationships of *lnK* (consistency index) and the non-Newtonian index *n* with the ultrasonic energy *Eu* are obtained by linear fitting of the data in reference [20], as shown in Figure 3. The microdimension correction parameters *a*_3_ and *b*_3_ were obtained by reference [19].

Therefore, the MU viscosity model for the ultrasonic micro-injection of PP is

(6) $$\eta_{\mathrm{micro}}=(\frac{-45.0388}{d}+0.819)\times e^{\left(10.033-0.003\times E_{u}\right)}{\dot{\gamma }}^{\left(\frac{-3.053}{E_{u}}-0.65\right)}$$

The simulated height of the micropart was subsequently obtained, to calculate the filling capacity according to the formula $f=(H_{s}/H)\times 100\%$, where *Hs* is the average value of the height of the rectangle injection molded part, and *H* is the micro-groove depth of the mold. The model parameters and process parameters of PP are shown in Table 3.

#### 3.2.2. Simulation of Pressure Difference

In order to verify the feasibility of the viscosity model for PS in this paper, a finite element model was established according to reference [13]. Based on the material properties of PS, the microscale flow was set as a generalized Newtonian fluid flow under isothermal conditions in the Fluent simulation. Equation (5) was imported into the preprocessing module of simulation software as a user-defined function to describe the polymer viscosity. The pressure difference obtained from the simulation was compared with that obtained from the experiment in [13] to verify the accuracy of the viscosity model.

The injection part was a rectangular thin plate with a thickness × length × width of 500 μm × 6.5 mm × 9.2 mm. In this model, *d* was set as 500 μm, and *Eu* was set as 50 W and 150 W. The initials temperature of PS (PG-383M, Zhenjiang Chimei, China) was 200 °C, the wall temperature of the mold was 25 °C. The inflow speed is set as the average flow velocity of the polymer in the reference [13] as shown in Table 4. The model parameters a1, b1, a2, and b2 were obtained by fitting the experimental data in reference [13], and the model parameters a3 and b3 were obtained by fitting the experimental data in reference [25]. The fitted values are listed in Table 4. The material chosen in the simulation was the same PS as that used in the experiment.

The MU viscosity model for PS is

(7) $$\eta_{\mathrm{micro}}=\left(1-\frac{25.194}{d}\right)\times e^{\left(11.809-0.01\times E_{u}\right)}{\dot{\gamma }}^{\left(0.4546+\frac{-14.498}{E_{U}}-1\right)}$$

The other parameters of model are detailed in Table 4. The pressure difference Δ*P* was defined as the average pressure difference between inlet and outlet of mold.

## 4. Results and Discussion

### 4.1. Validation of the MU Viscosity Model

#### 4.1.1. Filling Capacity of PP Ultrasonic Micro-Injection

**Figure 4:**
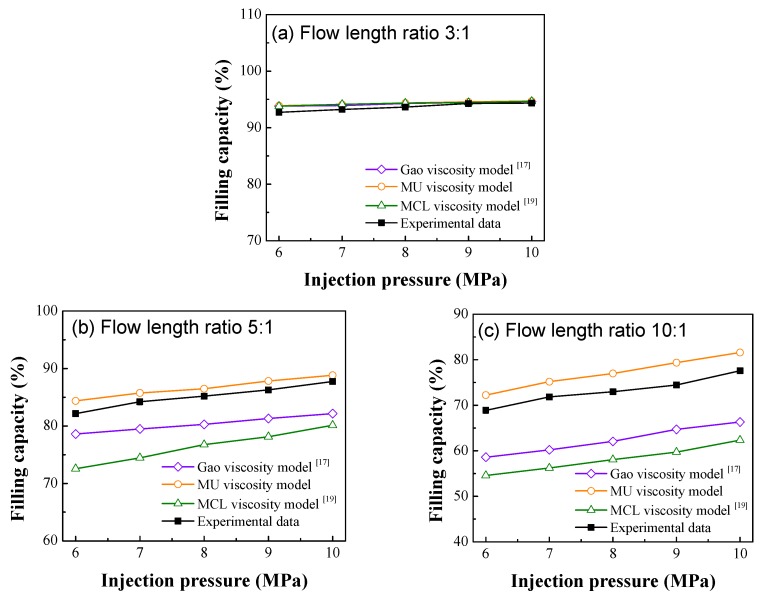
Simulation results and experimental results of microgroove filling capacities at different flow length ratios (**a**) flow length ratio 3:1; (**b**) flow length ratio 5:1; (**c**) flow length ratio 10:1.

The simulation results of the microgroove filling experiment using the MU viscosity model at different injection pressures were compared with the experimental results (Figure 4). In addition, the microgroove filling experiment was also simulated by using the ultrasonic viscosity model proposed by Gao et al. (Gao viscosity model) [17] and the MCL microviscosity model [19]. When the flow length ratio of the microgroove was 3:1 (Figure 4a), the simulated values from the three viscosity models were very close to each other and were only slightly different from the experimental values. The differences became more obvious at flow length ratios of 5:1 (Figure 4b) and 10:1 (Figure 4c). The maximum errors between the values simulated by the MU viscosity model and the experimental values were 2.7% and 4.9%, respectively, while the errors between the simulated values of the Gao viscosity model and the MCL microviscosity model were quite larger from the experimental values. The results indicate that the MU viscosity model can better characterize the rheological properties of the polymer during ultrasonic microinjection molding, especially when the microgroove flow length ratio is greater than 5:1. This is because the MU viscosity model takes into account the effects of the microstructure characteristic dimension and the ultrasonic energy. However, the values simulated by the MU viscosity model were a little greater than the experimental value. This is because the model assumes that the ultrasonic energy *E_U_* is completely absorbed by the material, while the actual ultrasonic energy is partly consumed by the work done to overcome the friction of the horn.

As the microstructure flow length ratio increased, the MCL microviscosity model [19] had larger errors and a lower simulated viscosity than those obtained by the Gao viscosity model [17] (Figure 4b,c). The results suggest that the viscosity reduction effect by ultrasonic vibration is greater than the microscale viscosity reduction effect during ultrasonic micro-injection molding, which affects the filling performance of the polymer melt.

#### 4.1.2. Pressure Differences in PS Micro-Injection Molding

**Figure 5:**
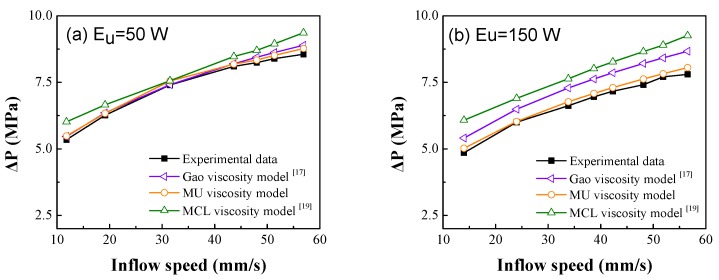
Pressure differences in ultrasonic microinjection at different inlet inflow speed (**a**) Ultrasonic vibration energy E_U_ = 50 W; (**b**) Ultrasonic vibration energy E_U_ =150 W.

The pressure difference experiment of PS micro-injection molding was performed to further examine the validity of the MU viscosity model. The MU viscosity model, the Gao viscosity model, and the MCL viscosity model were imported into the finite element simulation software Fluent as user-defined functions, while the physical and boundary conditions remained unchanged. The simulated pressure differences obtained by the MU viscosity model, the Gao viscosity model [17], and the MCL viscosity model [19] were compared with the experimental result [13] at different *E_U_* values (Figure 5).

At *E_U_* = 50 W, the simulated values of the MU viscosity model and the Gao viscosity model were relatively close to the experimental values. The simulated values of the MCL viscosity model had a large deviation from the experimental values because the MCL model does not consider the effect of ultrasonic vibration on viscosity. The more the vibration, the lower the viscosity, the better the polymer flow performance, and the smaller the pressure difference. Therefore, the simulated values obtained by the MCL model without considering ultrasonic vibration were larger than the experimental values. When the ultrasonic energy *E_U_* was increased to 150 W, the ultrasonic viscosity reduction effect increased. The maximum error of the MU viscosity model against the measured pressure difference was 3.2%, which was significantly smaller than that of the Gao viscosity model. This is because the MU model takes into account the change in the non-Newtonian index *n* under the influence of ultrasounds. Moreover, the MU model describes the microflow from the perspective of the characteristic dimension and molecular chain. The Gao viscosity model ignores the effect of microscopic size on viscosity and the change of *n* under ultrasonic energy [26] which virtually magnifies the power exponent term and the consistency index term in the model, thereby increasing the deviation between the simulated viscosity and the actual viscosity. Therefore, the MU model can more accurately reflect the actual microflow characteristics under ultrasonic energy.

### 4.2. Ultrasonic Microinjection Molding Process Analysis with Different Flow Length Ratios

#### 4.2.1. Injection Pressure

**Figure 6:**
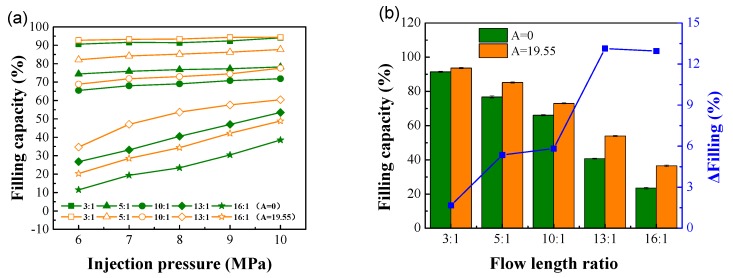
Effect of injection pressure on the filling capacity. (**a**) Filling capacity at different injection pressures; (**b**) Filling capacity at different flow length ratios and an injection pressure of 8 MPa.

**Figure 7:**
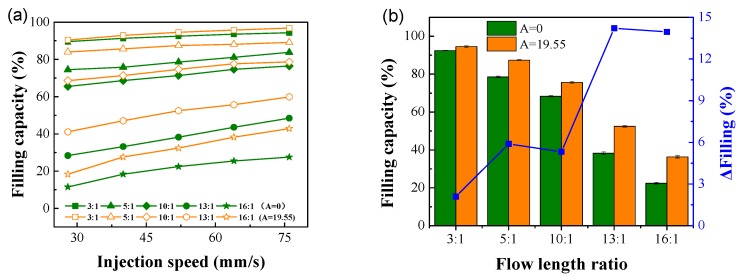
Effect of injection speed on filling capacity. (**a**) Filling capacity at different injection speeds; (**b**) Filling capacity at different flow length ratios and an injection speed of 52 mm/s.

The filling capacities of conventional micro-injection molding and ultrasonic micro-injection molding at different injection pressures and different flow length ratios are shown in Figure 6. The injection speed was set as 40 mm/s. A = 0 means conventional micro-injection molding, and A = 19.55 means ultrasonic-assisted vibration micro-injection with an amplitude of 19.55 μm. Figure 7a shows that when the microgroove flow length ratios were 3:1, the polymer melt could easily and completely fill the microgrooves. The increase in the microstructure filling capacity was not obvious with the increase in the injection pressure. However, when the flow length ratio was high (exceeding 5:1), the filling capacity increased significantly as the injection pressure increased.

In addition, as the flow length ratio increased, the filling difficulty increased, and the filling capacity gradually decreased (Figure 6b). When the flow length ratio was low (3:1), the effect of ultrasonic-assisted vibration on the filling capacity was not obvious. When the flow length ratio was greater than 5:1, the effect of ultrasonic-assisted vibration on the filling capacity was obvious, especially when the flow length ratio was high (greater than 13:1), the ultrasonic-assisted vibration can increase the filling capacity by 13.4%. The results indicate that the application of ultrasonic energy can promote the flow properties of the polymer molecules, reduce the viscosity, and thereby increase the filling capability.

#### 4.2.2. Injection Speed

The filling capacities of conventional micro-injection molding and ultrasonic micro-injection molding at an injection pressure of 7 MPa, different injection speeds, and different flow length ratios are shown in Figure 7. Figure 7a shows that the filling capacity increased as the injection speed increased. The higher the flow length ratio, the more significant the filling capacity increment as the injection speed changed. This is because of the microscale effect. The higher the injection speed, the stronger the shear effect of the polymer melt as it passes through the microstructure. The intensified shear promotes intermolecular friction in the polymer and increases the melt temperature. As a result, the viscosity reduction effect is more obvious, which leads to a better polymer flow performance and a more significant increase in the filling capacity.

The filling capacities of conventional micro-injection molding and ultrasonic micro-injection molding at an injection speed of 52 mm/s and different flow length ratios are shown in Figure 7b. Similar to the case when the injection pressure was changed, the increase in the filling capacity was most significant at high flow length ratios of 13:1 and 16:1. When the flow length ratio was 3:1, it is more difficult to increase the filling capacity when a high filling capacity has already been achieved because the mold does not have an exhaust system. Therefore, the increase in the filling capacity was not obvious after the application of ultrasonic energy. When the flow length ratio increased, the flow time of the polymer in the microstructure increased, and the degree of heat exchange between the polymer and the mold increased. Consequently, the cooling of the polymer was accelerated, and the filling capacity decreased while the mold cavity space increased. When ultrasonic energy was applied, the cooling rate of the polymer slowed down because of the ultrasonic thermal effect, and the filling capacity increased significantly. Therefore, the ability of ultrasounds to increase the filling capacity was more obvious at high flow length ratios.

#### 4.2.3. Ultrasonic Amplitude

**Figure 8:**
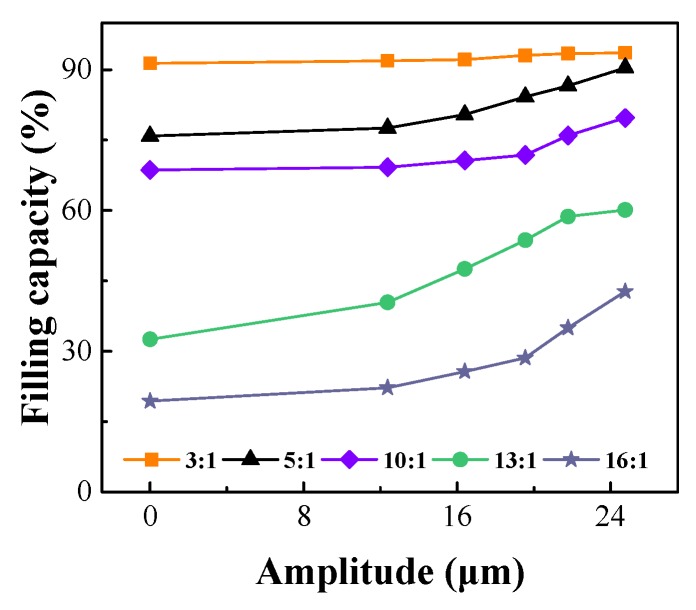
Effect of the ultrasonic amplitude on the filling capacity (injection pressure 7 MPa, injection speed 40 mm/s).

The filling capacities at different ultrasonic amplitudes and different flow length ratios are shown in Figure 8. When the flow length ratio was 3:1, the ultrasonic amplitude had little effect on the filling capacity, and the filling capacity was basically constant as the amplitude increased. At all other flow length ratios, the filling capacity increased as the amplitude increased because when the ultrasonic amplitude increased, the ultrasonic energy *Eu* increased, the polymer viscosity decreased, and the filling capacity increased.

## 5. Conclusions

(1) The MU viscosity model was established based on the ultrasonic energy, the characteristic microdimension, and the molecular chain length. The microgroove filling experiment with PP and the pressure difference experiment with PS were performed to examine the validity and accuracy of the MU viscosity model. The accuracy of the model was high when the microgroove flow length ratio was greater than 5:1 and the ultrasonic energy was 150 W. The maximum error was 4.2%.
(2) Ultrasonic microinjection molding experiments were performed using microgrooves with different flow length ratios. The results show that the larger the flow length ratio and the larger the amplitude, the more obvious the increase in the filling capacity by ultrasonic-assisted vibration. Especially in the case of high flow length ratios (greater than 13:1), ultrasonic vibration can increase the filling capacity by 13.4%.

## Figures and Tables

**Table 1:** Cross-sectional dimensions of the rectangular microgrooves.

Flow Length Ratio			Design Dimension		Actual Dimension	
	Width (μm)	Depth (μm)	Width (μm)	Standard Deviation	Depth (μm)	Standard Deviation
3:1	150	450	161.022	1.661	418.909	1.063
5:1	200	1000	216.880	1.154	970.873	1.539
10:1	150	1500	168.466	1.269	1433.480	1.194

**Table 2:** Experimental parameters.

Parameter	Injection Pressure (MPa)	Injection Speed (mm/s)	Ultrasound Amplitude (μm)
Group A	6, 7, 8, 9, 10	40	19.55
Group B	7	28, 40, 52, 64, 76	19.55
Group C	7	40	0, 16.40, 19.55, 24.76

**Table 3:** MU viscosity model parameters and process parameters for PP.

Parameter	Value	Parameter	Value
Consistency correction parameter *a*_1_	10.033	Ultrasonic amplitude *A* (μm)	19.55
Consistency correction parameter *b*_1_	−0.003	Frequency *f* (KHz)	38.9
Non-Newtonian index correction parameter *a*_2_	0.350	Injection pressure (MPa)	6,7,8,9,10
Non-Newtonian index correction parameter *b*_2_	−3.053	Injection speed (mm/s)	40
Microdimension correction parameter [19] *a*_3_	−22.66	Mode temperature (°C)	25
Microdimension correction parameter [19] *b*_3_	0.819	Viscous flow activation energy [21] E_η_ (kj·mol)	48.1
Chain length [22] *le* (nm)	2.18	Specific heat capacity [23] C (kj/(kg·°C))	1926
Sound absorption coefficient [24] α	0.112	Density [23] ρ (kg/m^3^)	910
Ultrasound velocity [24] *u* (m/s)	1582	Thermo conductivity [23] W/(m·K)	0.138
Ultrasonic active area S (mm^2^)	25.13		

**Table 4:** MU viscosity model parameters and rheological parameters for PS.

Parameter	Value	Parameter	Value
Consistency correction parameter *a*_1_	11.809	Ultrasonic amplitude *A* (μm)	20.30, 35.16
Consistency correction parameter *b*_1_	−0.01	Frequency *f* (KHz)	20
Non-Newtonian index correction parameter *a*_2_	0.455	Ultrasonic energy *E_U_* (W)	50, 150
Non-Newtonian index correction parameter *b*_2_	−14.498	Injection pressure (MPa)	6,7,8,9,10
Microdimension correction parameter a_3_	−12.597	Injection speed (mm/s)	40
Microdimension correction parameter b_3_	1.000	Mode temperature (°C)	25
Chain length [22] *le* (nm)	2.00	Viscous flow activation energy [21] E_η_ (kj·mol)	92.1
Sound absorption coefficient [24] α	0.0803	Specific heat capacity [23] C (j/(kg·°C))	1389
Ultrasound velocity [24] *u* (m/s)	2320	Density [23] ρ (kg/m^3^)	1050
Ultrasonic active area S (mm^2^)	78.54	Thermo conductivity [23] W/(m·K)	0.095
